# Medical College and Hospital Notes

**Published:** 1898-02

**Authors:** 


### Medical College and Hospital Notes.

THE board of directors of the German hospital has lately published its first annual report, which contains some interesting items. Practical relief work was begun on December 14, 1896, when the free dispensary was opened at 621 East Genesee street. The number of patients who were treated at the dispensary without charge, from December 14, 1896, to September 30, 1897, was: surgery, 469; diseases of women, 327; diseases of children, 178; diseases of eye, nose and throat, 332; skin and other diseases, 263; nervous diseases, 26; total, 1,900.

Another important event in the history of the German hospital has been the donation of a lot on Jefferson street, near Genesee, for a hospital site, by the heirs of the Gerhard Lang estate, acting through Edwin G. S. Miller. This gift was accompanied by a promise of $5,000 from the same source for the hospital. Plans for a three-story building, with all modern appliances, have been drawn, and it is expected that the hospital will be in operation before the close of the present year.

The present officers of the hospital are: President, Charles H. North; vice-president, Dr. Charles H. W. Auel; treasurer, Jacob Lang; secretary, M. J. Chemnitz; financial secretary, Charles Duchmann; corresponding secretary, George F. Lehmann; directors, Messrs. North, Auel, Chemnitz, Duchmann, Lang and E. G. S. Miller, Ottomar Reinecke and William Simon.

---

A PUBLIC hospital is about to be established at North Tonawanda. A citizens' and doctors' committee was appointed to meet on Friday evening, January 21, 1898, for the purpose of formulating plans to be followed. The Committee is made up as follows: William Gombert, Joshua S. Bliss, L. T. Payne, J. S. Thompson, John Bollier, William B. Kerr, Frank Batt, Mayor McKeen, Levant R. Van Dervoort, Dr. H. C. Leonhardt, Dr. A. T. Leonard, Dr. J. H. Helwig, Dr. C. R. Reagan, Dr. A. J. Martin.

The plan is not to make the hospital a charge upon the municipality, but to establish and maintain it by subscriptions from corporations, private individuals, church and charity organisations.

---

Rush Medical College, of Chicago, is preparing to affiliate with the University of Chicago. The University trustees have announced the terms on which the medical college will be received and if these are accepted the relationship will be consummated June 1, 1898. It is certainly desirable that medical colleges of standing should make University affiliations whenever possible.

